# *Leptospermum scoparium* Essential Oil: Antibacterial Properties, Cyto/Genotoxic Effects, Antioxidant Activity, and Wound-Healing Potential In Vitro

**DOI:** 10.3390/molecules31183304

**Published:** 2026-09-17

**Authors:** Mária Bučková, Andrea Puškárová, Dominika Galová, Marek Straka, Magda Suchánková, Domenico Pangallo, Monika Šramková, Katarína Kozics

**Affiliations:** 1 Institute of Molecular Biology, Slovak Academy of Sciences, Dúbravská Cesta 21, 84551 Bratislava, Slovakia; maria.buckova@savba.sk (M.B.); andrea.puskarova@savba.sk (A.P.); dominika.galova@savba.sk (D.G.); 2 Institute of Microbiology, Faculty of Medicine, Comenius University and University Hospital Bratislava, 81108 Bratislava, Slovakia; marek.straka@fmed.uniba.sk (M.S.);; 3 Cancer Research Institute, Biomedical Research Center, Slovak Academy of Sciences, Dúbravská Cesta 9, 84505 Bratislava, Slovakia; monika.sramkova@savba.sk (M.Š.); katarina.kozics@savba.sk (K.K.)

**Keywords:** *L. scoparium* essential oil, antibacterial activity, human keratinocytes HaCaT, cyto/genotoxic effects, total antioxidant status, wound-healing potential

## Abstract

This study evaluated *Leptospermum scoparium*, (manuka) essential oil (LSEO, for its antibacterial activity, cyto/genotoxicity, antioxidant potential, and its effects on wound closure, reflecting the growing interest in natural compounds with antimicrobial and potential wound-related cellular effects. Analyses were performed on the human keratinocyte cell line HaCaT. The genotoxic effect and the scratch assay of LSEO on human keratinocytes are described for the first time. The results revealed that LSEO did not induce significant DNA damage in vitro after 24 h of treatment. Moreover, treating HaCaT cells with LSEO increased the total antioxidant status level and enhanced wound closure at a concentration of 0.008 µg/mL. Additionally, the inhibitory effect of LSEO against several multidrug-resistant bacterial strains, including *Pseudomonas aeruginosa*, *Proteus vulgaris*, *Escherichia coli*, *Enterobacter cloacae*, and *Staphylococcus aureus*, was investigated. LSEO exhibited strong antibacterial activity, with inhibition zones ranging from 25 to 44 mm. The minimal inhibitory concentration and minimal bactericidal concentration of LSEO ranged from 0.10 to 0.5 μg/mL and from 0.25 to 0.75 μg/mL, respectively. The findings indicate that LSEO exhibits antibacterial activity against the tested multidrug-resistant bacterial isolates and may warrant further investigation as a potential antimicrobial agent.

## 1. Introduction

Human skin serves as the first line of defense as a barrier against environmental factors such as pathogens. When the skin is injured, the bacteria from the human skin can cause an infection and impede the healing process [1]. In particular, infections caused by multidrug-resistant bacteria, such as *Pseudomonas aeruginosa*, *Citrobacter koseri*, *Proteus vulgaris*, *Klebsiella pneumoniae*, *Acinetobacter baumannii*, and *Staphylococcus aureus*, pose an increasing threat to human health [2,3,4]. Over the past several decades, the emergence of multidrug-resistant bacteria has become a major global concern, complicating the selection of effective treatments for patients with skin infections [5,6]. With the growing use of broad-spectrum antibiotics, increasing antimicrobial resistance has complicated the selection of effective treatments for patients with skin infections [7,8,9]. This situation has further highlighted the need to explore new therapeutic approaches, particularly those based on natural medicinal products, when evaluating their efficacy in wound healing. Over recent decades, the mechanisms of wound repair have been studied extensively [10], allowing us to understand the role of keratinocytes in the initiation of migration and proliferation from the wound margins [11]. Inflammation and wound healing are associated with an increase in reactive oxygen species (ROS) and reactive nitrogen species (RNS), which can damage DNA [12,13].

Essential oils (EOs), formed by aromatic plants as secondary metabolites, are volatile, natural, complex compounds characterized by a strong odor [14]. EOs have a long history of traditional use and have gained popularity, particularly for skincare purposes [14]. EO from *Leptospermum scoparium* (LSEO, manuka—a shrub of the *Myrtaceae* family) has demonstrated various antibacterial [15,16,17,18,19], antifungal [20,21,22], antiparasitic [23], antiviral [24], and anti-inflammatory effects [22]. Many studies related to manuka, however, are focused on manuka honey; less is known regarding the uses of the LSEO [16]. Some research has been performed on the antimicrobial effects of LSEO, though studies of the effects of LSEO on eukaryotic cells (on human skin cells) are rare [25]. It has been shown that high concentrations of EOs in products intended for the treatment of skin disorders or wound healing may exhibit toxic effects on epithelial cells [26]. Therefore, evaluating the cytotoxic, genotoxic, and antioxidant properties of EOs is crucial for optimizing their safe and effective application in dermatological therapies.

In this study, we evaluated the antibacterial activity of LSEO against multidrug-resistant (MDR) bacterial strains *Pseudomonas aeruginosa*, *Proteus vulgaris*, *Escherichia coli*, *Enterobacter cloacae*, and *Staphylococcus aureus* obtained from patients with skin infections. Particularly, the minimum inhibitory concentration (MIC) and minimum bactericidal concentration (MBC) were assessed. Additionally, we investigated the cytotoxic and genotoxic effects, as well as the total antioxidant capacity and wound-healing potential, of this EO in vitro using the normal human keratinocyte (HaCaT) cell line. The wound-healing process was simulated by creating a linear scratch in a confluent monolayer of keratinocytes. Overall, this work aimed to assess the antibacterial activity, cytotoxicity, genotoxicity, antioxidant status, and wound-closure effects of LSEO under in vitro experimental conditions.

## 2. Results

### 2.1. Antimicrobial Susceptibility Testing to Antibiotics

The resistance to antibiotics of the target clinical bacterial strains: *P. aeruginosa*, *P. vulgaris*, *E. coli*, *E. cloacae*, and *S. aureus* is shown in Table 1. The results show that the strains isolated from wound infections were highly resistant to most of the beta-lactams, cephalosporins, and aminoglycosides.

### 2.2. Antibacterial Activity of LSEO

The antibacterial activity of LSEO varied among the tested MDR bacterial strains (Table 2). The largest zone of inhibition was observed against *S. aureus* KLI 434/2216 (44 mm), indicating high susceptibility to LSEO. Isolate *P. vulgaris* KLI 696/12 also exhibited strong inhibition (38 mm), followed by *E. coli* KLI 743 (35 mm) and *E. cloacae* RES 667 (28 mm). The lowest inhibition zone was recorded for *P. aeruginosa* KLI 696/2 (25 mm), suggesting a comparatively higher resistance to LSEO. Overall, the results demonstrate that LSEO possesses broad-spectrum antibacterial activity. The inhibition zones of LSEO were higher than reference antibiotic comparator cefuroxime (5 ± 2.0 mm).

**Table 2:** Zone of inhibition of LSEO used in the study (mm).

Bacteria	LSEO	CXM
*P. aeruginosa* KLI 696/2	25 ± 0.054	5 ± 0.02
*P. vulgaris* KLI 696/12	38 ± 0.052	7 ± 0.03
*E. coli* KLI 743	35 ± 0.068	7 ± 0.02
*E. cloacae* RES 667	28 ± 0.044	5 ± 0.04
*S. aureus* KLI 434/2216	44 ± 0.064	5 ± 0.08

LSEO: *L. scoparium* essential oil; CXM: cefuroxime. Data are represented as means ± SD of three independent experiments. MIC and MBC of the tested LSEO against 5 MDR bacterial strains are presented in Table 3.

**Table 3:** MIC and MBC; values of LSEO against tested MDR bacteria (µg/mL).

Bacterial Strains	LSEO	
	MIC	MBC
*P. aeruginosa* KLI 696/2	0.50	0.75
*P. vulgaris* KLI 696/12	0.25	0.25
*E. coli* KLI 743	0.25	0.50
*E. cloacae* RES 667	0.50	0.75
*S. aureus* KLI 434/2216	0.10	0.25

The results demonstrate that *S. aureus* KLI 434/2216 exhibited the highest sensitivity, with the lowest MIC (0.10 µg/mL) and MBC (0.25 µg/mL) values. *P. vulgaris* KLI 696/12 also showed strong susceptibility, with equal MIC and MBC values of 0.25 µg/mL, indicating a potent bactericidal effect. Isolate *E. coli* KLI 743 required slightly higher concentrations (MIC 0.25, MBC 0.50 µg/mL) for growth inhibition and killing. In contrast, *P. aeruginosa* KLI 696/2 and *E. cloacae* RES 667 were less sensitive, both showing MIC and MBC values of 0.50 and 0.75 µg/mL, respectively. Overall, the data indicate that LSEO possesses broad-spectrum antibacterial activity, with variable efficacy depending on the bacterial species.

### 2.3. The Antioxidant Activity of LSEO

**Figure 1:**
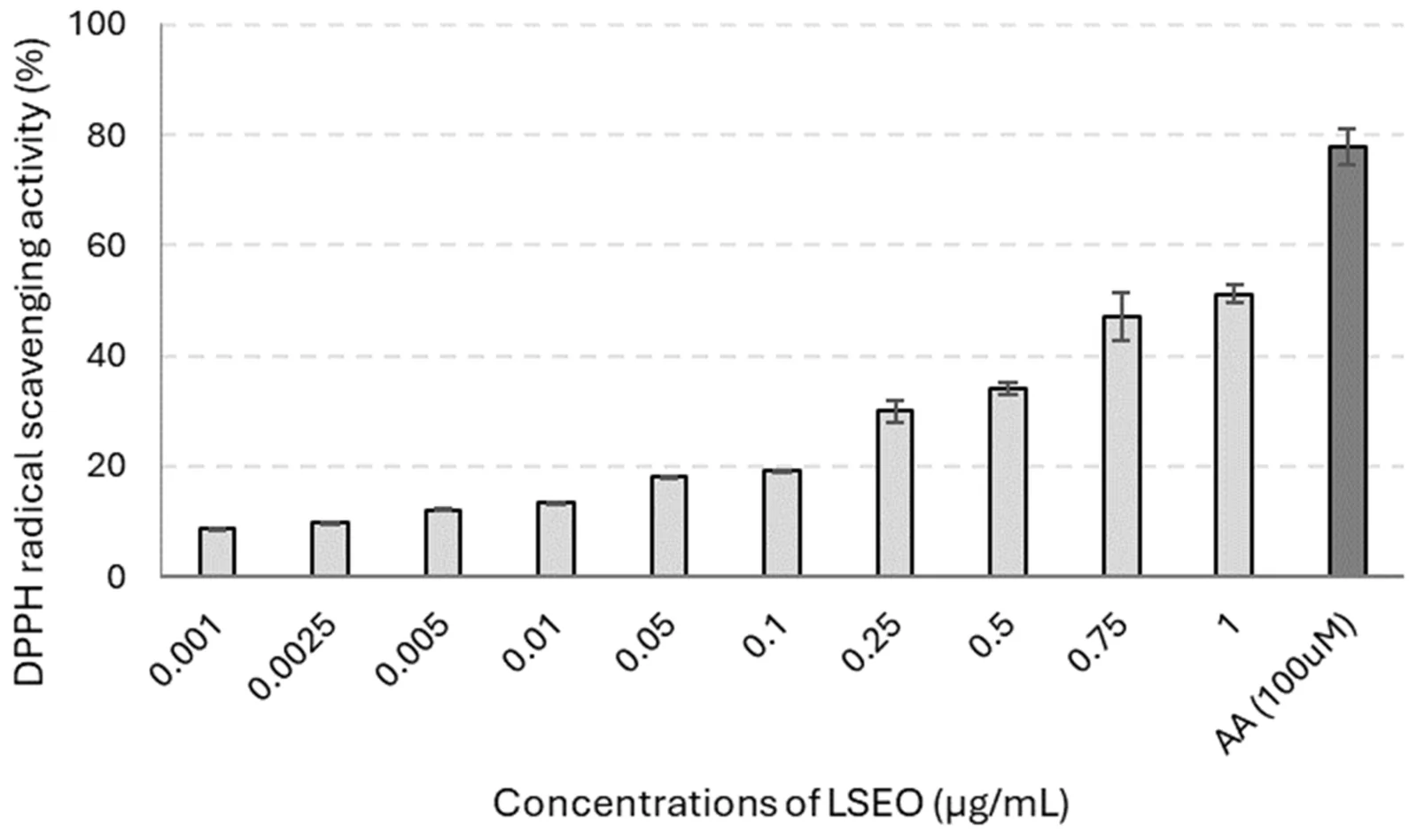
Antioxidant activity of LSEO determined with the DPPH assay. Data represent the means ± SD of three independent experiments. Ascorbic acid (AA) was used as a positive control (100 µM).

The results of the DPPH assay, as shown in Figure 1, demonstrate that LSEO exhibited concentration-dependent DPPH radical-scavenging activity. While the activity was relatively low at 0.001 µg/mL (8.7%), it increased markedly at higher concentrations, reaching 47% and 51% at 0.75 and 1 µg/mL, respectively. Although lower than the activity of the positive control AA (78%), the scavenging capacity observed at the highest LSEO concentrations demonstrates a substantial antioxidant potential.

### 2.4. Cytotoxic and DNA-Damaging Effects of LSEO

**Figure 2:**
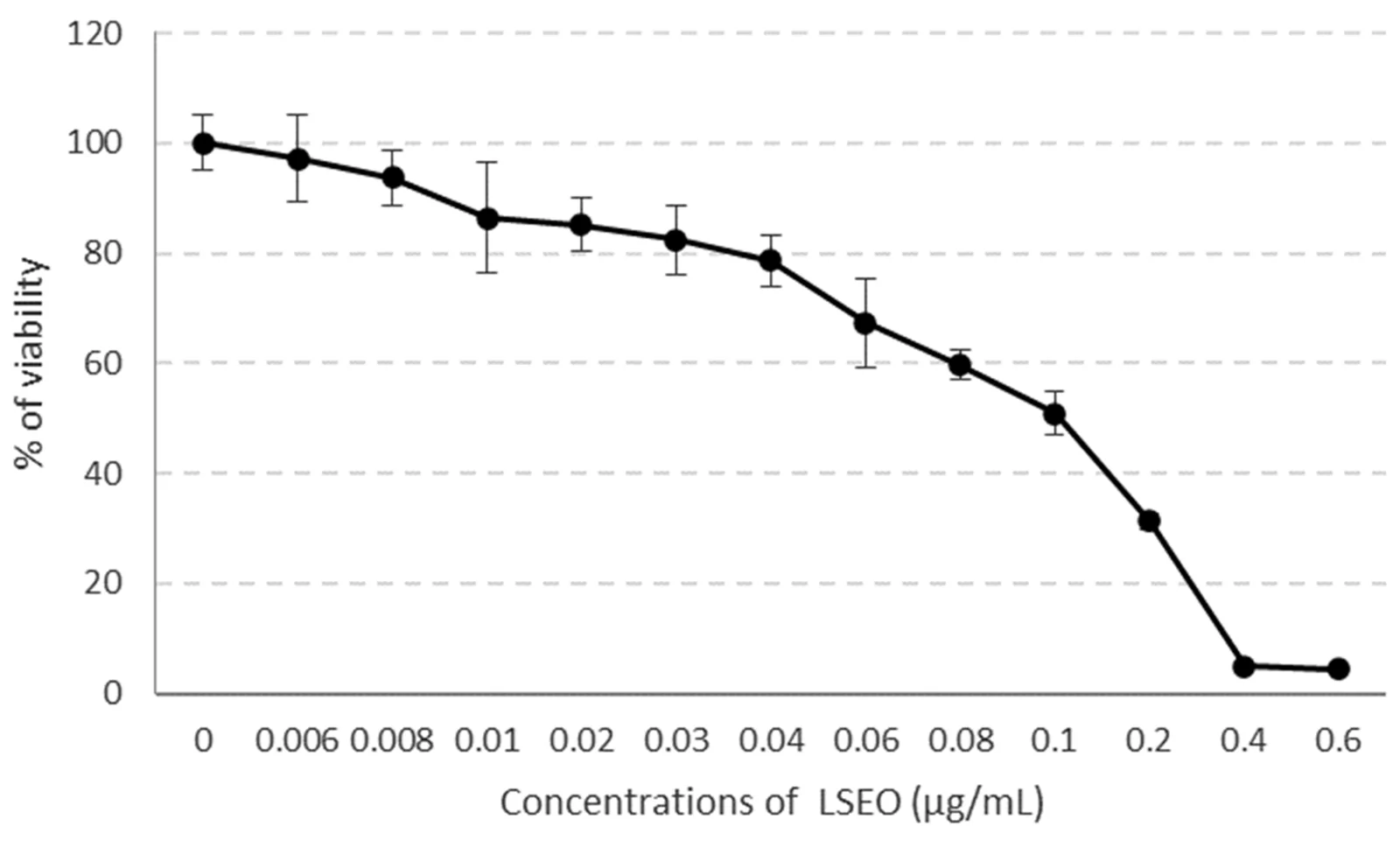
Cytotoxic effects of LSEO on the HaCaT cell lines after 24 h of treatment. Data are represented as means ± SD of three independent experiments.

The cytotoxicity of LSEO was assessed in HaCaT cells following 24 h exposure to various concentrations (0–0.6 µg/mL) using the MTT assay. The results are presented in Figure 2. The IC50 value was approximately 0.1 µg/mL and was estimated by interpolation from the concentration–response curve obtained from the MTT assay.

**Figure 3:**
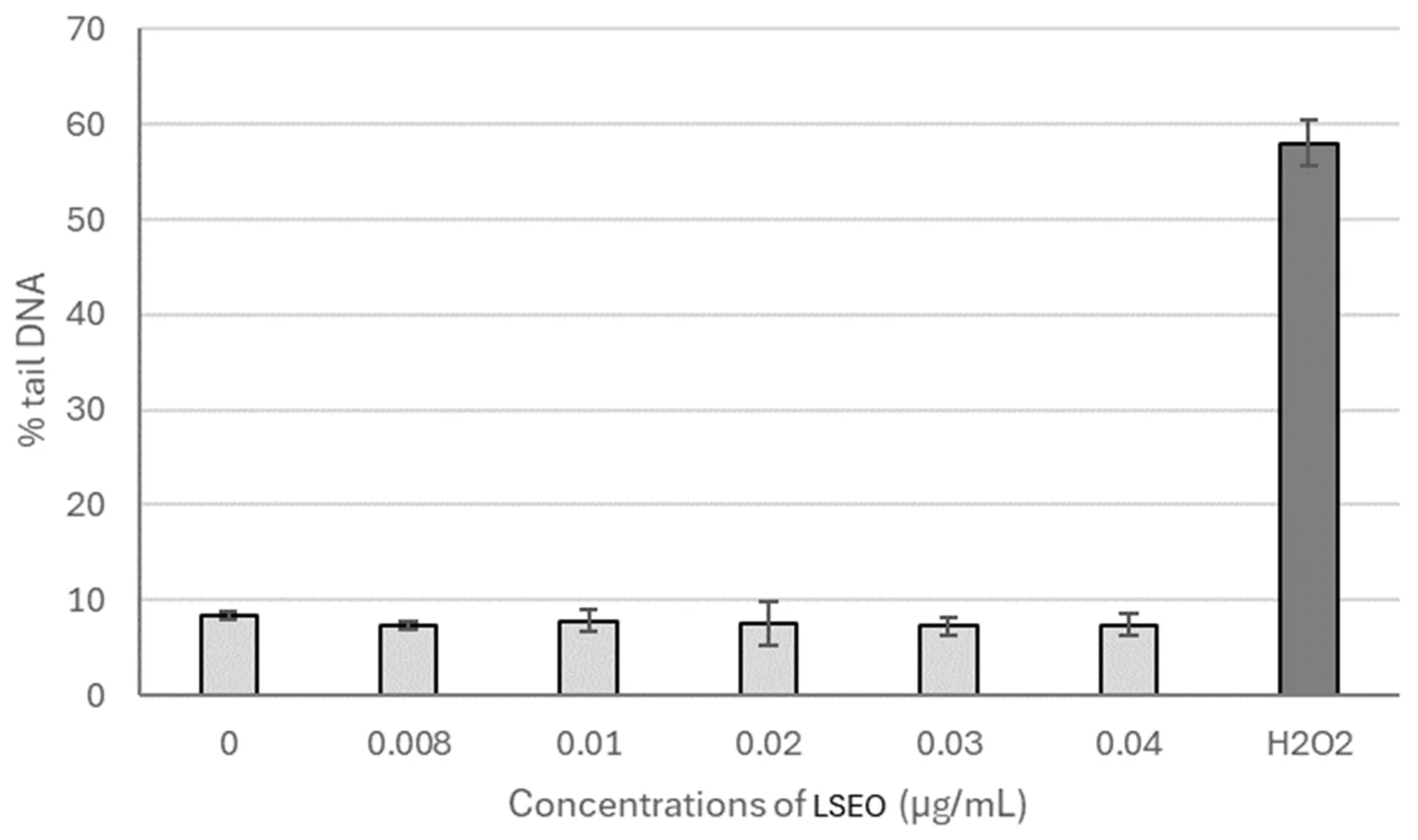
The levels of DNA single-strand breaks (% of tail DNA) in HaCaT cells after exposure to LSEO for 24 h. Data represent means ± SD of three independent experiments. Hydrogen peroxide was used as a positive control (300 µmol/L).

The genotoxic effects of LSEO were studied at concentrations of 0.008–0.04 µg/mL, selected from the concentration–response cytotoxicity curve to represent concentrations corresponding approximately to IC20. DNA strand breaks in HaCaT cells were evaluated using the comet assay and expressed as the % of tail DNA. Compared to untreated control cells, LSEO did not induce significant DNA damage (Figure 3) at any studied concentration.

### 2.5. Total Antioxidant Status Level of LSEO

TAS levels for the LSEO are presented in Table 4. Our results indicate that 24 h treatment of HaCaT cells with LSEO influenced TAS levels in a dose-dependent manner. In all tested concentrations of LSEO, TAS levels were significantly elevated compared to the negative control.

### 2.6. Wound-Healing Potential of LSEO

The repeated-measures ANOVA revealed significant effects of both time (F (22, 20.3) = 69.27, *p* < 0.0001) and LSEO concentration (F (3, 26.1) = 69.79, *p* < 0.0001) on wound closure. Wound healing progressed over time in all groups, with the control and the lowest concentration (0.008 µg/mL) showing the most rapid closure. Higher concentrations (0.01 and 0.02 µg/mL) were associated with significantly slower wound closure. Pairwise comparisons of least squares mean confirmed significant differences between all concentrations (*p* < 0.001), with the highest concentration (0.02 µg/mL) showing the least wound closure (Table 5).

Pairwise differences in least squares means of wound closure (mm^2^/well) between treatment groups with varying concentrations of LSEO. Estimates represent the adjusted mean difference between groups, with associated standard errors, degrees of freedom (DF), *t*-values, and *p*-values. All comparisons were statistically significant (*p* < 0.05), indicating a dose-dependent effect of LSEO on wound healing.

**Figure 4:**
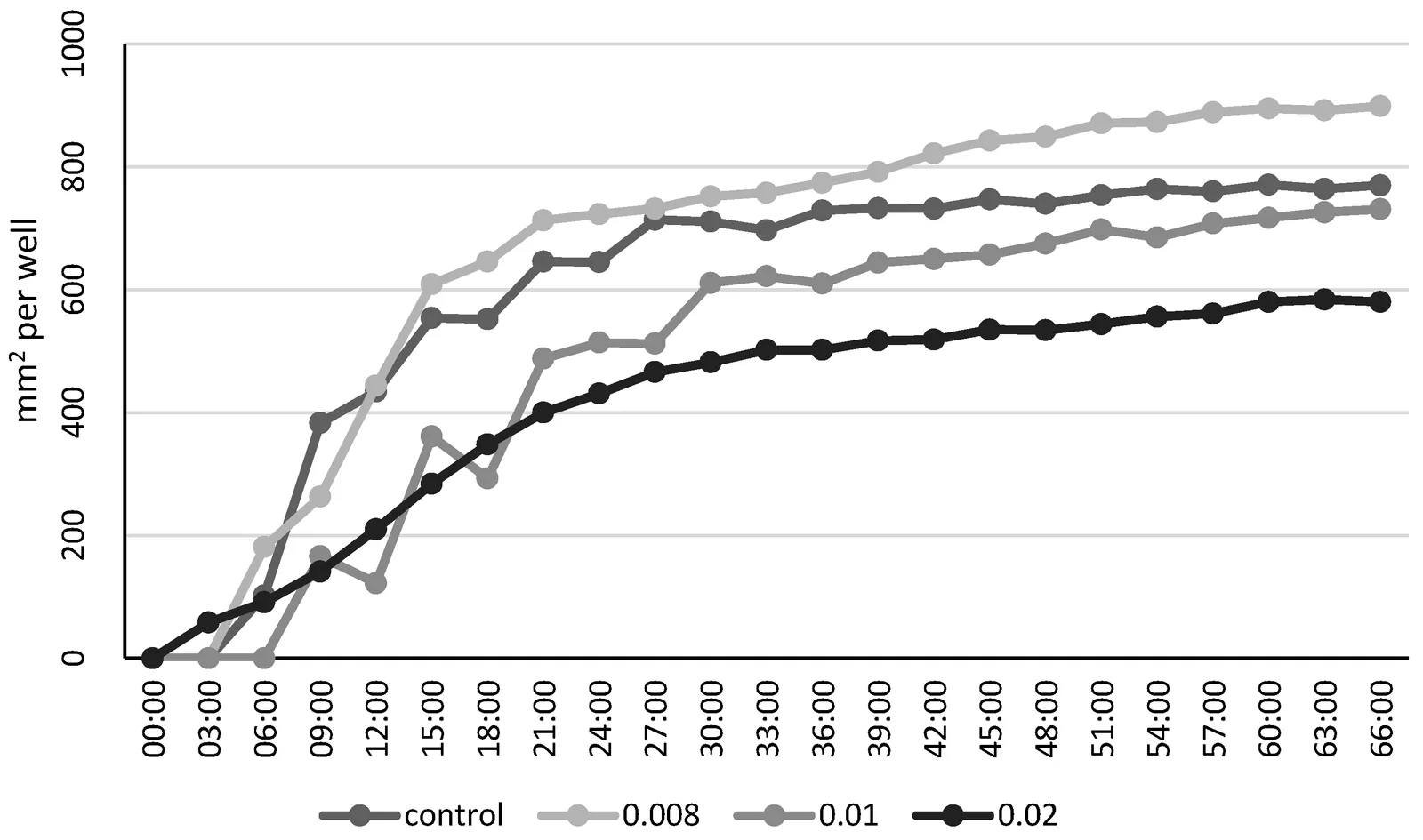
Wound healing over time across different LSEO concentrations. Wound closure (mm2/well) was measured over time in cell cultures treated with increasing concentrations of LSEO (0, 0.008, 0.01, and 0.02 µg/mL). Data represent model-estimated means from repeated-measures ANOVA using the SAS MIXED procedure. The control group and the lowest LSEO concentration showed the fastest wound closure, whereas higher LSEO concentrations were associated with slower wound closure.

Wound closure over time in cell cultures treated with different concentrations of LSEO. The *X*-axis represents time in hours (hh:mm), ranging from 00:00:00 to 66:00:00, and the *Y*-axis represents the measured wound closure area in mm^2^ per well. Data were obtained using the JULI^TM^ Stage live cell imaging system (NanoEnTek, Seoul, Republic of Korea). Each curve represents the average wound closure for a specific treatment group: control (medium only), and LSEO at concentrations of 0.008 µg/mL, 0.01 µg/mL, and 0.02 µg/mL. The lowest concentration (0.008 µg/mL) showed the most rapid wound closure, followed by the control group, while higher concentrations delayed healing (Figure 4).

## 3. Discussion

The present study demonstrates that LSEO exhibits antibacterial activity against MDR bacterial strains isolated from wound infections, including *P. aeruginosa*, *P. vulgaris*, *E. coli*, *E. cloacae*, and *S. aureus*. Pathogenic bacteria are increasing and becoming MDR due to irregular and inappropriate use of antibiotics [27]. The antibiotic resistance profiles of the clinical isolates in this study showed that most strains were clearly MDR. Despite this resistance, LSEO demonstrated inhibitory effects against all tested bacteria, with activity observed from concentrations as low as 0.15 µg/mL. Our results, regarding the antibacterial activities of LSEO, confirmed the previous findings of Choi et al. [28], which assayed this EO against methicillin-resistant *S. aureus* (MRSA). Similarly, Chen et al. [22] showed the antibacterial susceptibility to LSEO of *S. aureus*, *Streptococcus sobrinus*, *Streptococcus mutans*, and *E. coli*. Moreover, Song et al. [29] used EO from manuka in combination with Tris-EDTA against a total of 53 Gram-negative isolates (*P. aeruginosa*, *E. coli*, *K. pneumoniae ssp. pneumoniae*, and *P. mirabilis*). The antibacterial activity of LSEO is reported to be associated with the presence of β-triketones [30]. Studies also showed that the inhibition effect of LSEO is attributed to the disruption of the cytoplasmic membrane of the Gram-positive bacteria, whereas the resistance of Gram-negative bacteria is due to the lipopolysaccharides in their structure [31,32]. This explains the observed variability in susceptibility across the tested strains, with Gram-positive *S. aureus* being most sensitive and Gram-negative *P. aeruginosa* being least sensitive. LSEO inhibited the five tested drug-resistant clinical isolates in vitro. These findings justify further preclinical investigation but do not establish that LSEO is an alternative or adjunct to antibiotics in patients.

The antioxidant potential of LSEO has attracted significant scientific interest due to its high content of bioactive compounds, particularly phenolics and flavonoids. In this study, the antioxidant potential of the LSEO samples was evaluated through the DPPH radical scavenging assays. The results of the DPPH test exhibited that LSEO had a potent concentration-dependent DPPH radical-scavenging activity. Similar results were also reported by Kaur et al. [33], who studied the potential of LSEO as a natural antioxidant in high-fat meat products. The reason might be that the antioxidant activity of LSEO is due to sesquiterpene compounds, as Kwon et al. [25] reported. In their study, when individual components in LSEO were tested for their antioxidant potential, only c-terpinene and terpinen-4-ol showed antioxidant activity [20]. Previous studies have reported antioxidant-related effects of *Leptospermum scoparium* extracts and oil, although the reported activity may vary considerably depending on the plant material, extraction method, and chemical composition of the oil [16,20,25].

In recent years, several studies have investigated the antimicrobial properties of LSEO. However, the available knowledge regarding its effects on eukaryotic cells remains limited. In this study, effects such as cytotoxicity, genotoxicity, and overall antioxidant status of LSEO on a human keratinocyte cell line were determined. The cytotoxic effect on the LSEO of HaCaT cells was evaluated using the MTT assay, and the results showed that the 24 h treatment with LSEO affected cell viability in a dose-dependent manner. The IC_50_ value was determined to be 0.1 µg/mL. To our knowledge, relatively few studies have investigated the cytotoxic effects of LSEO on human cells: Bass et al. [34], where LSEO treatment induced apoptosis in both normal fibroblasts (CUA-4) and fibrosarcoma cells (HT-1080). In comparison with our results, this study employed very high concentrations (100–500 µg/mL), whereas 100 µg/mL was not yet cytotoxic to the cell lines examined. A possible explanation may lie in the different botanical origin of the manuka material studied. Also, while fibroblasts are classified as skin cells, this study investigated LSEO’s effect on keratinocytes, which show a different level of susceptibility to EOs. Moreover, our previous long-standing research of various EOs showed that cytotoxic effects have typically been observed in the range of 0.008–2 µg/mL on various cell lines [3,35,36,37]. In addition to the cytotoxic effect of LSEO, its possible genotoxic effect on the HaCaT cell line was also evaluated. To avoid false positivity in the comet assay due to the cytotoxic effect of LSEO, cells were treated at a concentration of approximately IC_20_. Our results showed no significant increase in DNA strand breaks compared to untreated controls.

Oxidative stress is an imbalance between the production of ROS and the body’s ability to neutralize them. Antioxidants help reduce oxidative stress by scavenging ROS and preventing cellular damage. Our results showed that the 24 h treatment of HaCaT cells with LSEO significantly affected TAS levels in a dose-dependent manner. A recent study showed that adding LSEO to high-fat meat (wagyu beef) significantly reduced lipid oxidation, suggesting that this oil can act as a natural antioxidant in food systems [33]. A skin-model study found that topical application of LSEO prevented UV-B–induced oxidative skin damage and photoaging in mice. The authors attribute the effect to the antioxidant constituents of the oil (notably γ-terpinene and terpinen-4-ol), which likely scavenge ROS [25]. Many studies focus on MH rather than LSEO. For example, in vitro and in vivo experiments with MH demonstrated reduced oxidative damage (lower levels of lipid peroxidation, DNA damage, improved antioxidant enzyme activity), particularly under inflammatory or oxidative challenge [38,39,40].

Wound healing is a complex process that involves the control of homeostasis, inflammation, proliferation, formation, and remodeling of new tissue [41]. In the present study, LSEO affected wound closure in the HaCaT cell model in a concentration-dependent manner. The lowest tested concentration (0.008 µg/mL) resulted in faster wound closure compared with the untreated control, whereas higher concentrations (0.01 and 0.02 µg/mL) significantly delayed wound closure. These findings indicate that LSEO can influence wound closure under the experimental conditions used. However, since the scratch assay measures the overall reduction in the wound area, it does not allow the individual contribution of cell migration and cell proliferation to be distinguished. Therefore, the observed effect should not be interpreted as direct evidence of a specific mechanism of migration or proliferation. Further studies using dedicated migration and proliferation assays would be required to clarify the underlying mechanism. The statistical model confirmed significant effects of both time and treatment, with robust differences between all tested concentrations. The use of repeated-measures ANOVA allowed the temporal pattern of wound closure to be evaluated while accounting for repeated measurements within wells. Further studies are warranted to elucidate the molecular mechanisms underlying the observed effects and to validate these findings in more complex wound models or in vivo systems.

In the present study, LSEO affected wound closure in the HaCaT cell model in a concentration-dependent manner. The lowest tested concentration (0.008 µg/mL) resulted in faster wound closure compared with the untreated control, whereas higher concentrations (0.01 and 0.02 µg/mL) significantly delayed wound closure. These findings indicate that LSEO can influence wound closure under the experimental conditions used. However, since the scratch assay measures the overall reduction in the wound area, it does not allow the individual contributions of cell migration and cell proliferation to be distinguished. Therefore, the observed effect should not be interpreted as direct evidence of a specific mechanism of migration or proliferation. Similar concentration-dependent effects of essential oils on cellular responses have been reported in our previous studies

## 4. Materials and Methods

LSEO (100%) was obtained from doTERRA (Pleasant Grove, UT, USA). LSEO was weighed to determine the volume that comprised 10 mg and then diluted in DMSO. The human keratinocyte cell line HaCaT (T0020001) was purchased from AddexBio (San Diego, San Diego, CA, USA). Dulbecco’s Modified Eagle Medium (DMEM), fetal calf serum (FCS), and antibiotics (penicillin 100 U/mL; streptomycin 100 µg/mL) were purchased from Gibco BRL (Paisley, UK). 3-(4,5-Dimethyldiazol-2-yl)- 2,5-diphenyltetrazolium bromide (MTT), 2,2-diphenyl-1-picrylhydrazyl (DPPH), and methanol were purchased from Sigma–Aldrich (Merck KGaA, Darmstadt, Germany). Dimethyl sulfoxide (DMSO), Mueller-Hinton agar (MHA), and Mueller–Hinton broth (MHB) were purchased from Merck KGaA, Darmstadt, Germany.

The essential oil was extracted from the leaves of the plant by steam distillation. The chromatograms and the peak report of LSEO are described in the Supplementary Materials Section.

### 4.1. Bacterial Strains and Growth Conditions

Five MDR bacterial strains: *Pseudomonas aeruginosa* KLI 696/2, *Proteus vulgaris* KLI 696/12, *Escherichia coli* KLI 743, *Enterobacter cloacae* RES 667, *Staphylococcus aureus* KLI 434/2216 were isolated from the samples of patients of University Hospital Bratislava indicated for microbiological examination. These strains were identified within the routine microbiological diagnostics at University Hospital Bratislava [42]. Susceptibility testing for antibiotics (piperacillin, piperacillin/tazobactam, ceftazidime, cefepime, aztreonam, imipenem, meropenem, gentamicin, tobramycin, amikacin, ciprofloxacin, and ofloxacin) was evaluated using the disk-diffusion method. Susceptibility testing of these strains was performed and evaluated according to the EUCAST guidelines (http://www.eucast.org accessed on 13 January 2025).

Intermediate susceptibility was considered as resistant. MDR was defined as resistance to at least one agent in three or more antimicrobial classes [43].

### 4.2. Screening for Antibacterial Activity

A disc-diffusion assay was used to determine the growth inhibition of bacteria by LSEO. A single colony from an overnight bacterial culture plate was inoculated into 10 mL of the growth medium MHB. Culture tubes were shaken at 100 rpm and 37 °C until the 600 nm absorbance of the growth solution was greater than 1.0. Using a sterile swab, cultures were spread onto 37 °C agar plates. Sterile filter paper discs (5 mm Ø Whatman No.1) were gently pressed onto the surface of the agar plates, and 10 μL of LSEO (100%) was then pipetted onto the disc. A pure DMSO control was included in each test to ensure that bacterial growth was not inhibited by DMSO itself. The inhibition halos of LSEO were assessed according to Ponce et al. [44] as follows: not sensitive for a diameter less than 8 mm, sensitive for a diameter of 9–14 mm, very sensitive for a diameter of 15–19 mm, and extremely sensitive for a diameter larger than 20 mm. Cefuroxime (30 μg/disc; Merck KGaA, Darmstadt, Germany) was used in order to compare its antibacterial effect with that of LSEO. Plates were incubated for 24 h at 37 °C, and the diameter of the inhibition zones was measured in mm, including the diameter of the disc. Regarding cefuroxime, for areas smaller than 7 mm in diameter, the inhibitory effects were classified as not sensitive. The sensitivity to antibiotics was classified according to Eucast (http://www.eucast.org accessed on 3 March 2025).

### 4.3. Determination of MIC and MBC of LSEO

MIC and MBC of LSEO were determined using a broth microdilution method according to the Poaty et al. [45]. MIC was determined as the lowest concentration of LSEO that inhibited the visible growth of the tested bacteria. MBC was defined as the lowest concentration of LSEO at which the inoculum viability was reduced up to 99.9% or no apparent growth occurred. It was determined by subculturing from wells that inhibition of growth was visible on sterile MHA plates that do not contain LSEO. The plates were incubated at 37 °C for 24 h.

### 4.4. Radical-Scavenging Activity

The assay is based on the reduction in semi-persistent free radical, 2,2-diphenyl-1-picrylhydrazyl (DPPH•) by antioxidants and is monitored by the decolorization of the purple DPPH radical solution, to form pale yellow 2,2-diphenyl-1-picrylhydrazine (DPPH-H). The radical-scavenging activity of LSEO (0–1 µg/mL) was evaluated by DPPH assay according to Gupta [46] with some modifications. DPPH solution (100 µM) was prepared by dissolving DPPH radical in methanol. The reaction mixture (consisting of DPPH solution and LSEO) was added to a 48-well plate, shaken, and incubated in the dark for 30 min. After incubation, the absorbance of the resulting solution was measured at 517 nm using an xMark™ Microplate Spectrophotometer (Bio-Rad Laboratories, Inc., Hercules, CA, USA). Ascorbic acid was used as a positive control, and DPPH solution was used as a negative control. The antioxidant activity was evaluated using the following formula:

Scavenging of DPPH radicals (%) = (A_control_ − A_sample_/A_control_) × 100

where A_control_ is the absorbance of the negative control, absorbance of the blank DPPH•; A_sample_ is the absorbance of the tested compound.

### 4.5. Determination of Cytotoxicity

Cytotoxicity of LSEO was monitored by the colorimetric MTT assay. In this assay, the yellow tetrazolium salt 3-(4,5-dimethylthiazol-2-yl)-2,5-diphenyltetrazolium bromide (MTT) is reduced by mitochondrial enzymes of metabolically active cells to purple formazan and is spectrophotometrically quantified [47]. Briefly, 2 × 10^4^ HaCaT cells were seeded in 96-well plates and cultured in a complete DMEM medium. The LSEO in concentrations 0–0.6 µg/mL was then added, and the cells were incubated at 37 °C in a 5% CO_2_ atmosphere for 24 h. Next, cells were washed with phosphate-buffered saline (PBS) at the indicated time point, followed by incubation with 1 mg/mL MTT for 4 h. Then, the MTT was removed, and the formazan crystals were dissolved with DMSO for 30 min. Absorbance at a wavelength of 540 nm was measured using an xMark microplate Spectrophotometer (Bio-Rad Laboratories, Inc., Hercules, CA, USA), and background absorbance at 690 nm was subtracted. The results are presented as mean ± SD in quadruplicates from three independent experiments. The IC_50_ value was estimated by interpolation from the concentration–response curve.

### 4.6. Determination of Genotoxicity

Genotoxicity was determined by the alkaline comet assay (single-cell gel electrophoresis, SCGE), which allows for the detection of DNA breaks [48]. Cells were seeded in a 6-well plate (3 × 10^4^ cells/well) and treated with LSEO (0.008–0.04 µg/mL) for 24 h. Next, cells were washed with PBS, followed by trypsinization. Lysis was performed in a cooled solution consisting of 2.5 M NaCl, 100 mM Na2EDTA, 10 mM Tri-HCl (pH 10), and 1% Triton X-100 for one hour in the cold. The samples were transferred to an electrophoretic solution (300 mM NaOH, 1 mM Na2EDTA, pH > 13) in an electrophoretic apparatus and allowed to unwind for 30 min at 4 °C in the dark. Electrophoresis (19 V, 300 mA) was then performed for 20 min at 4 °C. Samples were neutralized by washing in a neutralization solution for 2 × 10 min (0.4 M Tris-HCl, pH 7.4). After the slides had dried, ethidium bromide (5 µg/mL) was applied. The slides were examined using a Zeiss Imager.Z2 fluorescence microscope (Carl Zeiss Microscopy GmbH, Jena, Germany), with computerized image analysis (Metafer 3.6, MetaSystems GmbH, Altlussheim, Germany). The percentage of DNA in the tail was used as a parameter for the measurement of DNA damage (DNA strand breaks). Five hundred comets were scored per sample in one electrophoresis run.

### 4.7. Determination of Total Antioxidant Status

The total antioxidant status (TAS) was determined by a chromogenic method (Randox Laboratories, Crumlin, Ireland) with minor adaptations. This methodology is based on the capacity to inhibit the formation of the ABTS+ radical cation (2,20-azino-di-[3-etylbenzotiazolin sulphonate]). Absorbance at a wavelength of 600 nm was measured using a spectrophotometer xMarkTM Microplate Spectrophotometer (Bio-Rad Laboratories, Inc., Hercules, CA, USA). As the positive control, ascorbic acid (10 µmol/L, Sigma–Aldrich) was used. Results are expressed as µmol of TAS per gram of protein (µmol/g prot). The protein concentrations were determined using the Bradford method [49].

### 4.8. Scratch Assay

Wound healing was assessed in vitro using a scratch assay on the HaCaT cell line. Cells in a density of 2.5 × 10^5^ were seeded on a Petri dish. After reaching 100% confluence, cells were scratched on each Petri dish with a pipette tip. After the scratch, cells were treated with LSEO for 24 h. Experimental groups included a control and three treatment groups with increasing concentrations of LSEO (0.008–0.02 µg/mL). The wound closure was quantified in mm^2^ per well over time using the JULI™ Stage live (NanoEnTek, Seoul, Republic of Korea) cell-imaging system.

### 4.9. Statistical Analysis

The results represent the mean from 3 to 5 experiments ± standard deviation (SD). The differences between the control and treated samples were tested for statistical significance using Student’s *t*-test (* *p* < 0.05; ** *p* < 0.01; *** *p* < 0.001). Because the datasets for the genotoxicity, antibacterial activity, and TAS level were normally distributed, the independent samples *t*-test was performed to test for significant differences between groups. Differences between more than two groups in wound healing were assessed by one-way analysis of variance (ANOVA) followed by the Bonferroni test if equal variances were assumed or Tamhane’s test if equal variances were not assumed. Differences with *p*  <  0.05 are considered to be statistically significant.

Statistical analysis of the scratch assay was performed using SAS Enterprise Guide version 8.3 (SAS Institute Inc., Cary, NC, USA), employing a repeated-measures ANOVA via the MIXED procedure. The model included fixed effects for time and treatment concentration, and random effects to account for repeated measures within wells. Model fit was evaluated using AIC, BIC, and likelihood ratio tests. Least squares means and pairwise comparisons were used to assess differences between treatment groups.

## 5. Conclusions

In this study, the antibacterial effect of LSEO on MDR strains of *Pseudomonas aeruginosa, Proteus vulgaris*, *Escherichia coli*, *Enterobacter cloacae*, and *Staphylococcus aureus* was evaluated. The cell viability and scratch assay results showed that LSEO is not cytotoxic and may serve as a promising candidate for tissue repair in wound healing. At the lowest tested concentration, LSEO enhanced wound closure in the HaCaT in vitro model, whereas higher concentrations delayed wound closure. Additional research steps are needed to verify the use of LSEO for in vivo treatment of skin. Clinical studies are needed to assess the potential for in vivo wound-healing efficacy.

## Figures and Tables

**Table 1:** Susceptibility to antibiotics of multidrug-resistant bacteria.

Bacteria	PEN	AMP	AMS	AMX	CXM	CTX	CAZ	CIP	TMS	TET
*P. aeruginosa* KLI 696/2	ND	ND	R	ND	R	R	I	I	R	R
*P. vulgaris* KLI 696/12	ND	R	R	ND	R	S	S	S	S	R
*E. coli* KLI 743	ND	R	R	ND	R	S	R	R	R	R
*E. cloacae* RES 667	ND	R	R	ND	R	R	S	S	S	S
*S. aureus* KLI 434/2216	R	R	R	R	R	R	ND	R	S	S

PEN: penicillin; AMP: ampicillin; AMS: ampicillin + sulbactam; AMX: amoxicillin; CXM: cefuroxime; CTX: cefotaxime; CAZ: ceftazidime; CIP: ciprofloxacin; TMS: trimethoprim + sulfamethoxazole; TET: tetracycline; R: resistant; S: susceptible; I: susceptible increased exposure; ND: not determined.

**Table 4:** TAS in HaCaT cells exposed to LSEO for 24 h.

Sample	Dose (µg/mL)	TAS (mmol/prot)
Control (−)	-	0.61 ± 0.02
Control (+)	-	4.54 ± 0.23
LSEO	0.008	0.76 ± 0.04 **
	0.01	0.85 ± 0.03 ***
	0.02	1.03 ± 0.08 **
	0.04	1.13 ± 0.04 ***

Data are presented as means ± SD of three independent experiments. ** *p* < 0.01; *** *p* < 0.001 indicate statistically significant differences compared to the negative control (Student’s *t*-test).

**Table 5:** Pairwise comparisons of the least squares means for wound closure between LSEO concentrations.

Differences of Least Squares Means						
Concentrations		Estimate	Standard Error	DF	*t*-Value	Pr > |*t*|
0	0.008	−6.6652	1.3033	16.9	−5.11	<0.0001
0	0.01	11.0052	1.5500	26.8	7.10	<0.0001
0	0.02	18.7722	1.8012	22.0	10.42	<0.0001
0.008	0.01	17.6704	1.7198	22.8	10.27	<0.0001
0.008	0.02	25.4374	1.9492	32.2	13.05	<0.0001
0.01	0.02	7.7670	2.1221	25.3	3.66	<0.0012

## Data Availability

Data are contained within this article.

## Supplementary Materials

The following supporting information can be downloaded at: https://www.mdpi.com/article/10.3390/molecules31183304/s1, Figure S1: The chromatograms and the peak report of LSEO.
