# Supplementary material for: Leptospermum scoparium Essential Oil: Antibacterial Properties, Cyto/Genotoxic Effects, Antioxidant Activity, and Wound-Healing Potential In Vitro
Source: Molecules. 2026 Sep 17;31(18):3304. doi: 10.3390/molecules31183304 (PMC13609292; doi:10.3390/molecules31183304)
Supplement: Supplementary file 1 [file molecules-31-03304-s001.zip › molecules-4479508-supplementary.pdf]

Aromatic Plant Research Center

We provide uncompromising
quality control for your products.

Customer : doTERRA International
Lot Number : 192334
Date Filled : 08/21/2019

Column : ZB5 (60 m length × 0.25 mm inner diameter × 0.25 μm film thickness)
Instrument : Shimadzu GCMS-QP2010 Ultra
Carrier gas : Helium 80 psi
Temperature ramp : 2 degrees celsius per minute up to 260-degrees celsius
Split ratio : 30:1
Sample preparation : 5%w/v solution with Dichloromethane.

Comments:
The analysis of this Manuka lot revealed no contaminants or adulteration.
The sample meets the expected chemical profile for authentic essential oils of Leptospermum scoparium.

Analysied by : Ambika Poudel
Reviewed by : Dr. Prabodh Satyal

CERTIFIED BY AROMATIC PLANT RESEARCH CENTER

© Copyright 2019 Aromatic Plant Research Center. All rights reserved. Any publishing, copying, use, dissemination, or distribution of this report, including online, without the express written permission of Aromatic Plant Research Center is strictly prohibited.

Aromatic Plant Research Center
230 N 1200 E STE 100
Lehi, UT 84043
www.aromaticplant.org

Manuka Essential Oil

Customer : doTERRA International

Lot Number : 192334

Date Filled : 08/21/2019

Chromatogram

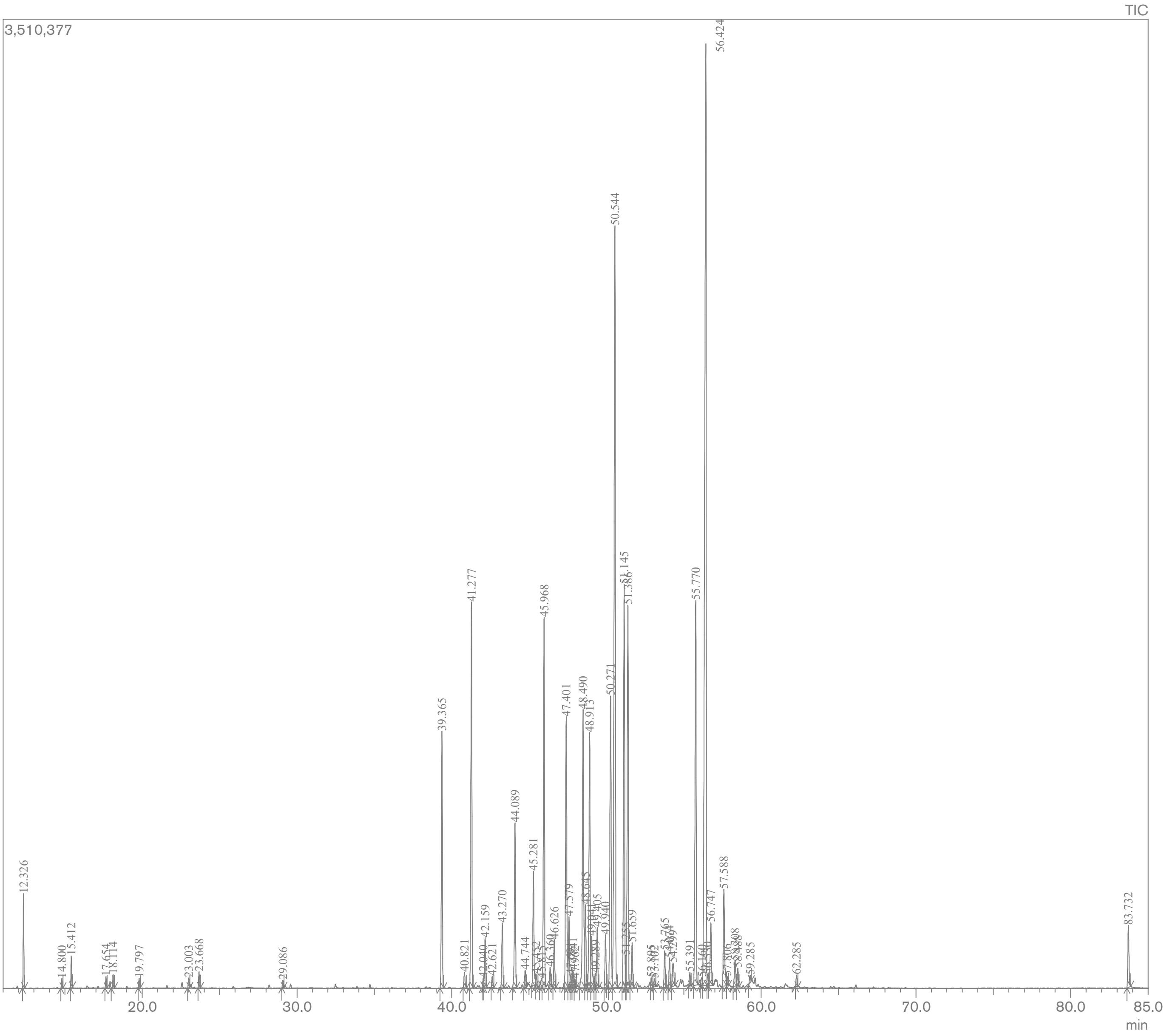

© Copyright 2019 Aromatic Plant Research Center. All rights reserved. Any publishing, copying, use, dissemination, or distribution of this report, including online, without the express written permission of Aromatic Plant Research Center is strictly prohibited.

Aromatic Plant Research Center
230 N 1200 E STE 100
Lehi, UT 84043
www.aromaticplant.org

APRC® Aromatic Plant Research Center

Peak Report

Peak#	R.Time	Name	Area%
1	12.326	alpha-Pinene	0.80
2	14.800	beta-Pinene	0.09
3	15.412	Myrcene	0.29
4	17.654	para-Cymene	0.11
5	18.114	1,8-Cineole	0.14
6	19.797	gamma-Terpinene	0.10
7	23.003	Isopentyl isovalerate	0.11
8	23.668	3-Methyl butanoate, 3-methyl-3-butenyl	0.17
9	29.086	2-methyl-1-Butyl tiglate	0.08
10	39.365	alpha-Cubebene	3.23
11	40.821	alpha-Ylangene	0.23
12	41.277	alpha-Copaene	5.20
13	42.040	beta-Cubebene	0.11
14	42.159	beta-Elemene	0.64
15	42.621	benzyl-Isovalerate	0.14
16	43.270	alpha-Gurjunene	0.86
17	44.089	beta-Caryophyllene	2.26
18	44.744	beta-Copaene	0.23
19	45.281	Aromadendrene	1.55
20	45.452	6,9-Guaiadiene	0.19
21	45.815	dihydro-Curcumene	0.16
22	45.968	trans-Murrola-3,5-diene	4.96
23	46.360	alpha-Humulene	0.25
24	46.626	Alloaromadendrene	0.62
25	47.401	cis-Cadina-1(6),4-diene	4.20
26	47.579	trans-Cadina-1(6),4-diene	0.94
27	47.691	gamma-Gurjunene	0.16
28	47.841	alpha-Amorphene	0.23
29	47.962	Germacrene D	0.11
30	48.490	beta-Selinene	4.56
31	48.645	trans-Muurola-4(14),5-diene	1.13
32	48.913	alpha-Selinene	3.68
33	49.041	alpha-Muurolene	0.79
34	49.289	epi-Zonarene	0.23
35	49.405	trans-trans-alpha-Farnesene	0.85
36	49.940	delta-Amorphene	0.70
37	50.271	delta-Cadinene	4.90
38	50.544	trans-Calamenene	12.71
39	51.145	trans-Cadine-1,4-diene	5.67
40	51.255	gamma-Calcorene	0.28
41	51.386	Flavesone	5.39
42	51.659	alpha-Calacorene	0.60
43	52.895	beta-Calcorene	0.13
44	53.102	Manuka sesquiterpenoid 1	0.10
45	53.765	Spathulenol	0.49
46	54.074	Caryophyllene oxide	0.39
47	54.299	Globulol	0.45
48	55.391	Ledol	0.16
49	55.770	iso-Leptospermone	5.52
50	56.160	Intermedeol isomer	0.13
51	56.424	Leptospermone	18.28
52	56.530	Eudesmol isomer	0.14
53	56.747	1-epi-Cubenol	1.01
54	57.588	Cubenol	1.58
55	57.806	delta-Cadinol	0.13
56	58.308	alpha-Eudesmol	0.45
57	58.488	alpha-Selin-11-en-4-ol	0.26
58	59.285	Cadalene	0.11
59	62.285	Muurol-5-en-4-one Isomer	0.14
60	83.732	Grandiflorone	0.87
			100.00

CERTIFIED BY AROMATIC PLANT RESEARCH CENTER

© Copyright 2019 Aromatic Plant Research Center. All rights reserved. Any publishing, copying, use, dissemination, or distribution of this report, including online, without the express written permission of Aromatic Plant Research Center is strictly prohibited.
